# Supplementary material for: Suppression of unwanted CRISPR-Cas9 editing by co-administration of catalytically inactivating truncated guide RNAs
Source: Nat Commun. 2020 Jun 1;11:2697. doi: 10.1038/s41467-020-16542-9 (PMC7264211; doi:10.1038/s41467-020-16542-9)
Supplement: Supplementary file 1 — Supplementary Information [file 41467_2020_16542_MOESM1_ESM.pdf]

Supplementary Information

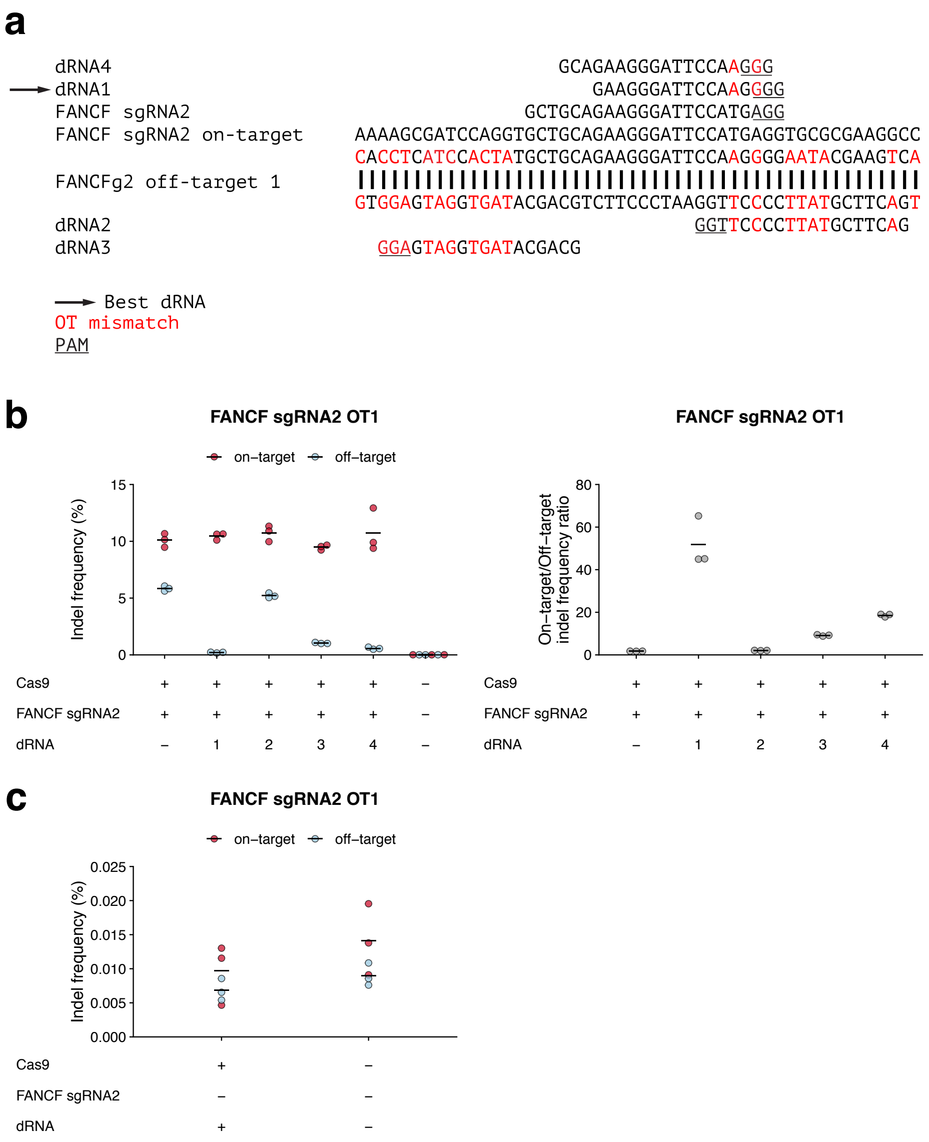

Supplementary Figure 1. *FANCF* dRNA1 does not promote Cas9-mediated editing. (a) Sequence alignment of *FANCF* sgRNA2, its on-target site, the most prominent off-target, off-target site 1 (OT1), and multiple dRNAs complementary to OT1. Black arrows indicate best dRNA, as determined by maximal off-target editing suppression with minimal on-target editing suppression. **(b)** Indel frequencies and specificity ratios (on-target/off-target indel frequency ratios) at the *FANCF* sgRNA2 on-target site and OT1 24 hours after transfection with Cas9, sgRNA, and various dRNAs. For conditions without dRNA, an equivalent amount of pMAX-GFP was substituted. **(c)** Indel frequencies at the *FANCF* sgRNA2 on-target and OT1 sites 24 hours after transfection with Cas9 and dRNA1 but no sgRNA. The predicted cut sites of dRNA1 are the same as *FANCF* sgRNA2. Indel frequencies for untransfected cells are shown as a control. Numbers denote dRNA identity, see **Supplementary Data Set 1**. Solid lines denote the mean of n = 3 biological replicates.

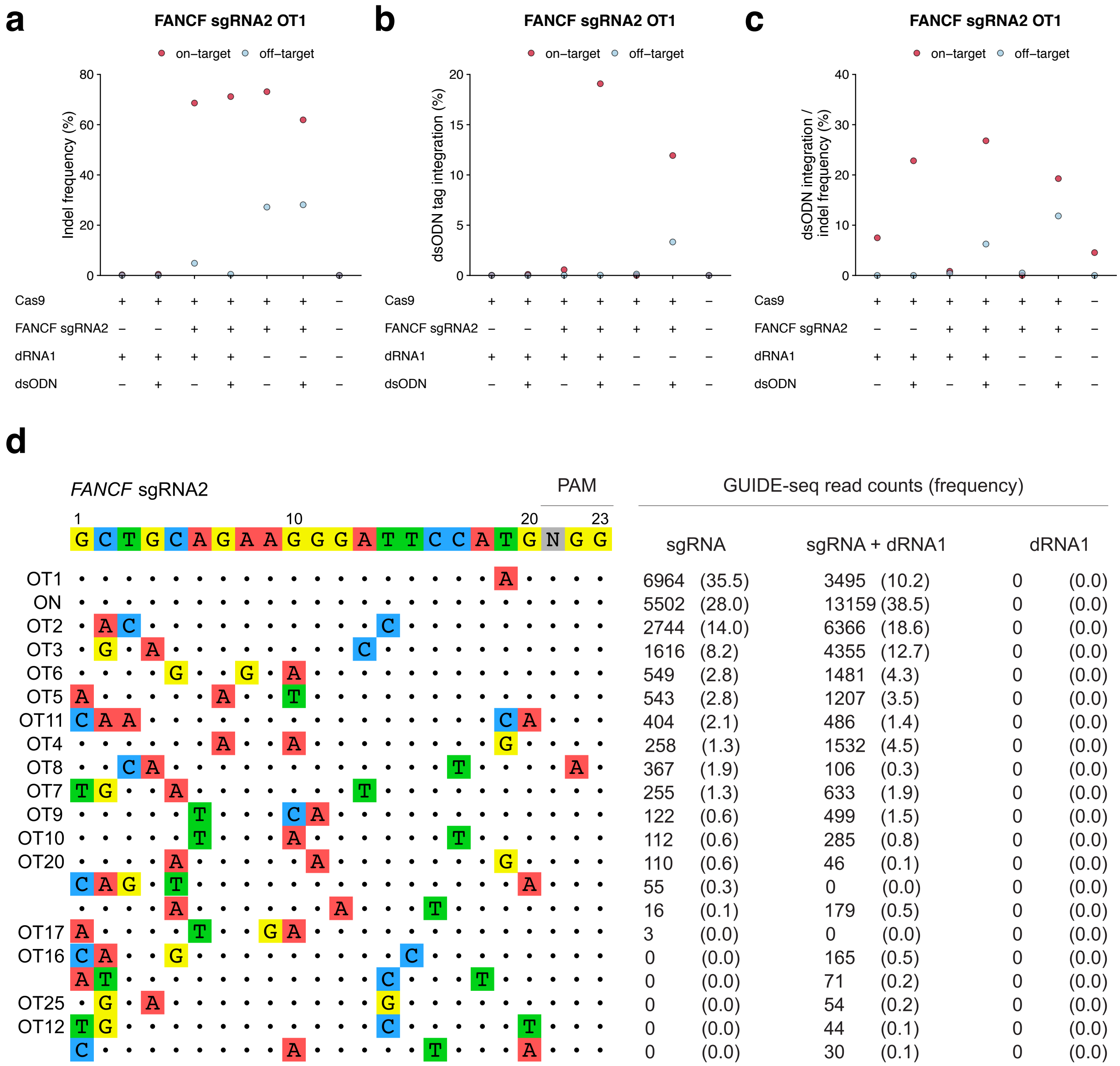

Supplementary Figure 2. Genome-wide assessment of DNA cleavage using dRNAs. (a) Indel frequency at *FANCF* sgRNA2 on-target site and OT1 96 hours after electroporation with plasmids encoding Cas9, sgRNA and dRNA1 in U2OS cells. **(b)** Integration of an end-protected double-stranded oligonucleotide (dsODN) 96 hours after electroporation with Cas9, sgRNA and dRNA1 in U2OS cells. **(c)** dsODN tag integration efficiency ratio (integration:indel) 96 hours after electroporation. Indel frequencies and dsODN tag integration for untransfected cells are shown as a control. **(d)** GUIDE-seq genome-wide specificity profiles for Cas9 paired with *FANCF* sgRNA2, *FANCF* dRNA1 or both allowing for up to 8 mismatches from on-target sequence. Mismatched positions in off-target sites are highlighted by color. Black dots indicate matched nucleotide to on-target site. GUIDE-seq counts and frequencies are shown to the right of the sequences. Off-target numbering system to the left of sequences adapted from Reference (18), Figure 2.

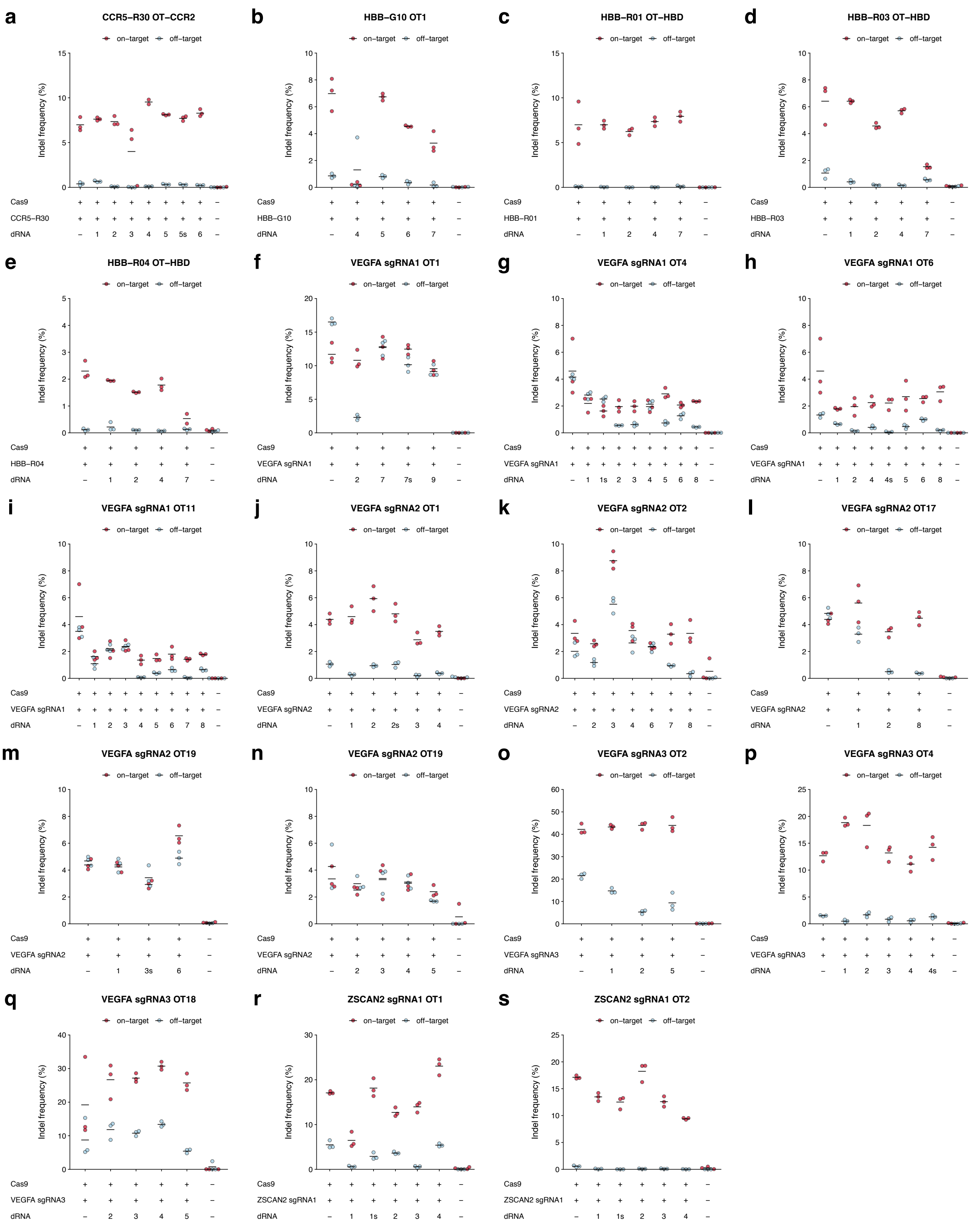

Supplementary Figure 3. dRNAs screened to increase the specificity ratios of 18 additional on-target/off-target pairs. On and off-target indel frequencies 24 hours after transfection with Cas9, sgRNA, and off-target specific dRNAs in HEK293T cells **(a)** *CCR5*-R30 OT (*CCR2*). **(b)**

HBB-G10 OT1. 4 additional dRNAs were screened, which are not shown here. **(c)** *HBB*-R01 OT (*HBD*). **(d)** *HBB*-R03 OT (HBD). **(e)** *HBB*-R04 OT (HBD). **(f)** *VEGFA* sgRNA1 OT1. **(g)** *VEGFA* sgRNA1 OT4. **(h)** *VEGFA* sgRNA1 OT6. **(i)** *VEGFA* sgRNA1 OT11. **(j)** *VEGFA* sgRNA2 OT1. **(k)** *VEGFA* sgRNA2 OT2. **(l)** *VEGFA* sgRNA2 OT17. **(m)** *VEGFA* sgRNA2 OT19 (dRNAs 1, 3s, and 6). **(n)** *VEGFA* sgRNA2 OT19 (dRNAs 2-5). **(o)** *VEGFA* sgRNA3 OT2. **(p)** *VEGFA* sgRNA3 OT4. **(q)** *VEGFA* sgRNA3 OT18. **(r)** *ZSCAN2* sgRNA1 OT1. **(s)** *ZSCAN2* sgRNA1 OT2. Indel frequencies for untransfected cells are shown as a control. Numbers denote dRNA identity, see **Supplementary Data Set 1**. Solid lines denote the mean of n = 3 biological replicates. OT = off-target.

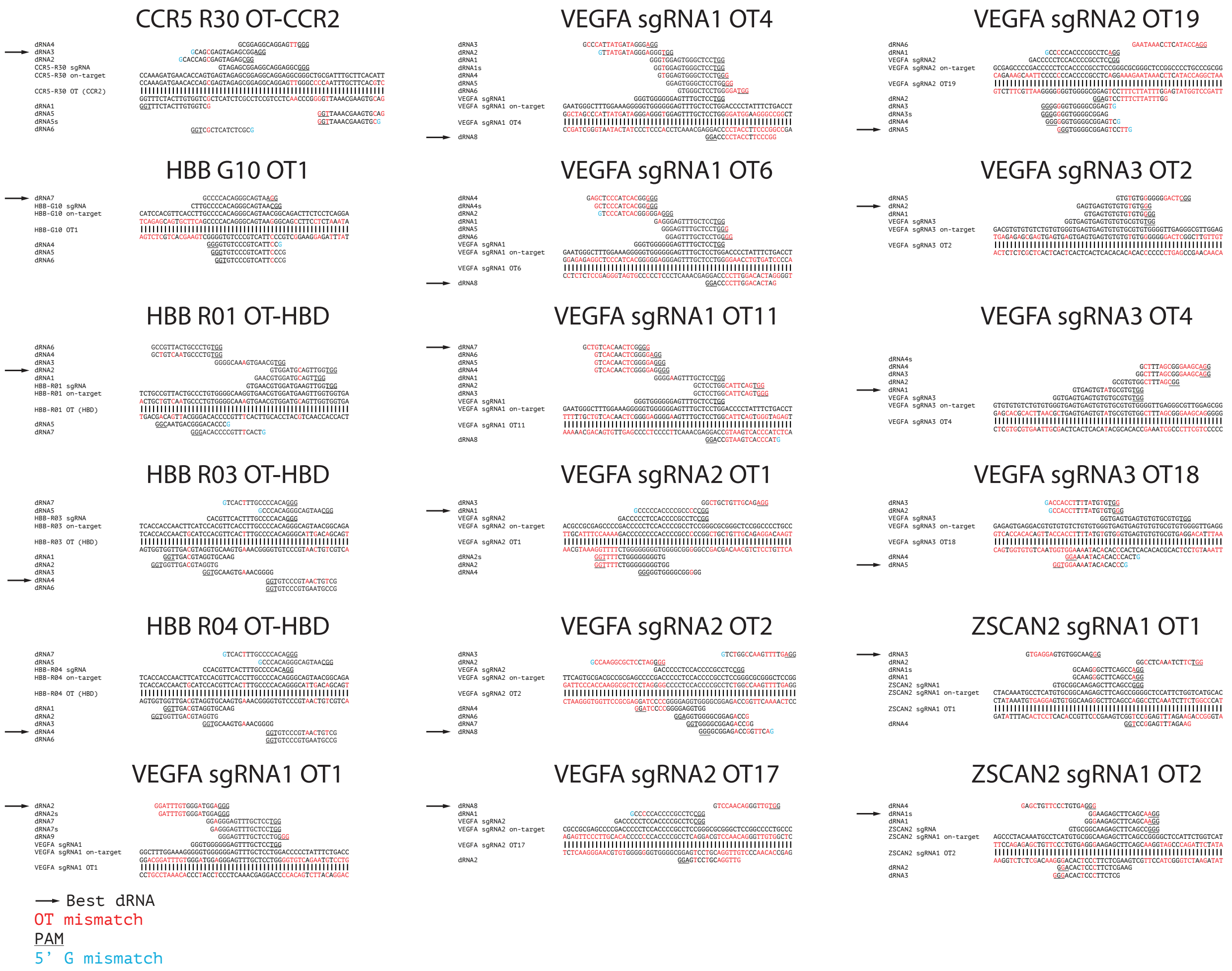

Supplementary Figure 4. Alignments of on-target sites and dRNAs to off-target sites. Sequence alignments for all off-targets used in this study (For *FANCF* sgRNA2, see Supplementary Figure 1) and their corresponding dRNAs and on-targets. Mismatches in the off-target and dRNA sequences relative to the on-target sequence are displayed in red. Mismatched 5' guanines in dRNAs are displayed in cyan. PAM sequences are underlined. Black arrows indicate best dRNA, as determined by maximal off-target editing suppression with minimal on-target editing suppression.

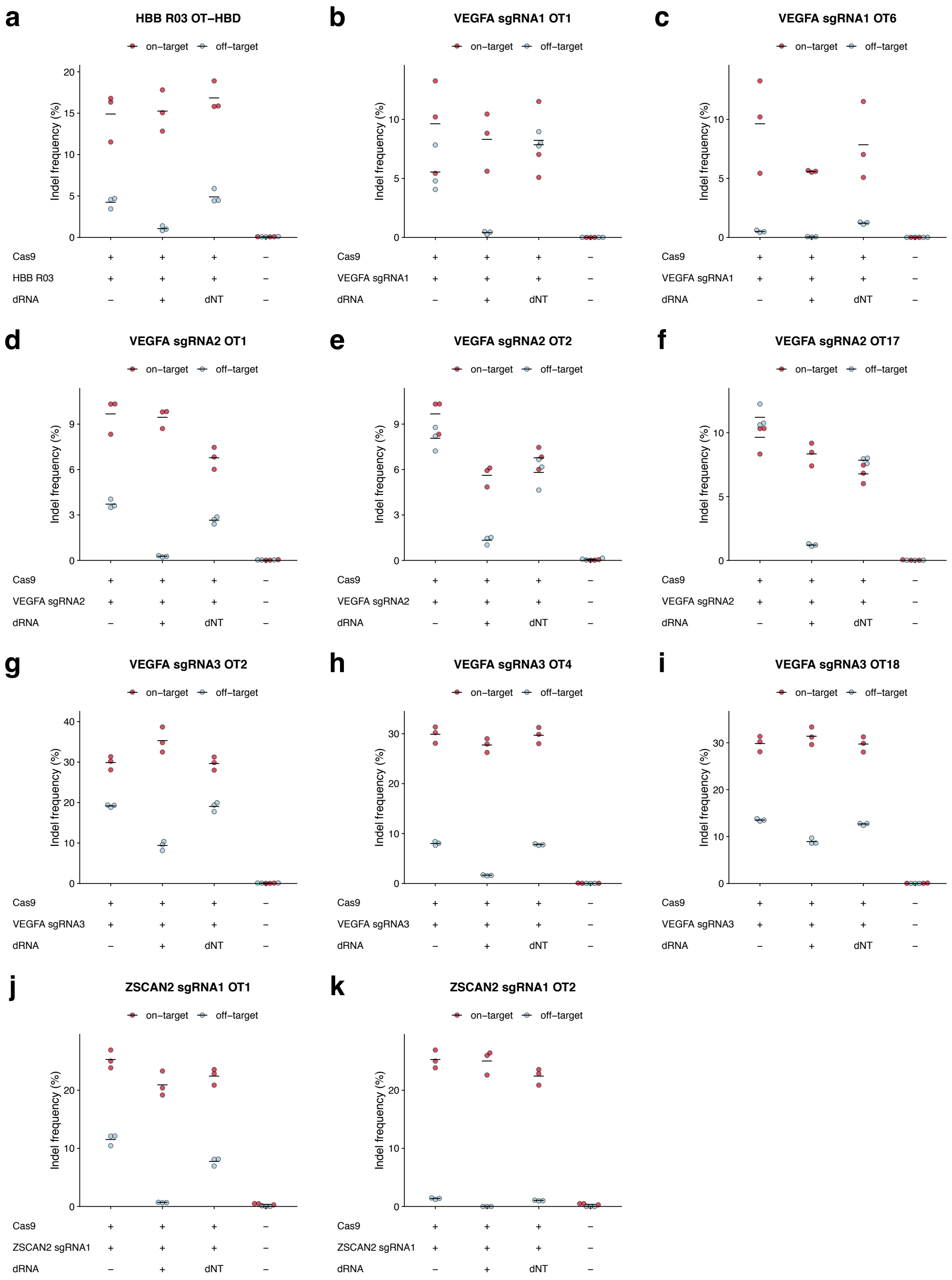

Supplementary Figure 5. Nontargeting dRNAs have minimal effects on on-target and off-target editing. Comparison of most effective dRNA for 12 different off-target loci with a nontargeting dRNA (dNT). Indel frequency of on-target and off-target loci 24 hours after transfection with Cas9, sgRNA, ± dRNA or nontargeting dRNA in HEK293T cells. Indel frequencies for untransfected cells are shown as a control. **(a)** *HBB* R03 OT-HBD. **(b)** *VEGFA* sgRNA1 OT1. **(c)** *VEGFA* sgRNA1 OT6. **(d)** *VEGFA* sgRNA2 OT1. **(e)** *VEGFA* sgRNA2 OT2. **(f)** *VEGFA* sgRNA2 OT17. **(g)** *VEGFA* sgRNA3 OT2. **(h)** *VEGFA* sgRNA3 OT4. **(i)** *VEGFA* sgRNA3 OT18. **(j)** *ZSCAN2* sgRNA1 OT1. **(k)** *ZSCAN2* sgRNA1 OT2. Numbers denote dRNA identity, see **Supplementary Data Set 1**. Solid lines denote the mean of n = 3 biological replicates. OT = off-target.

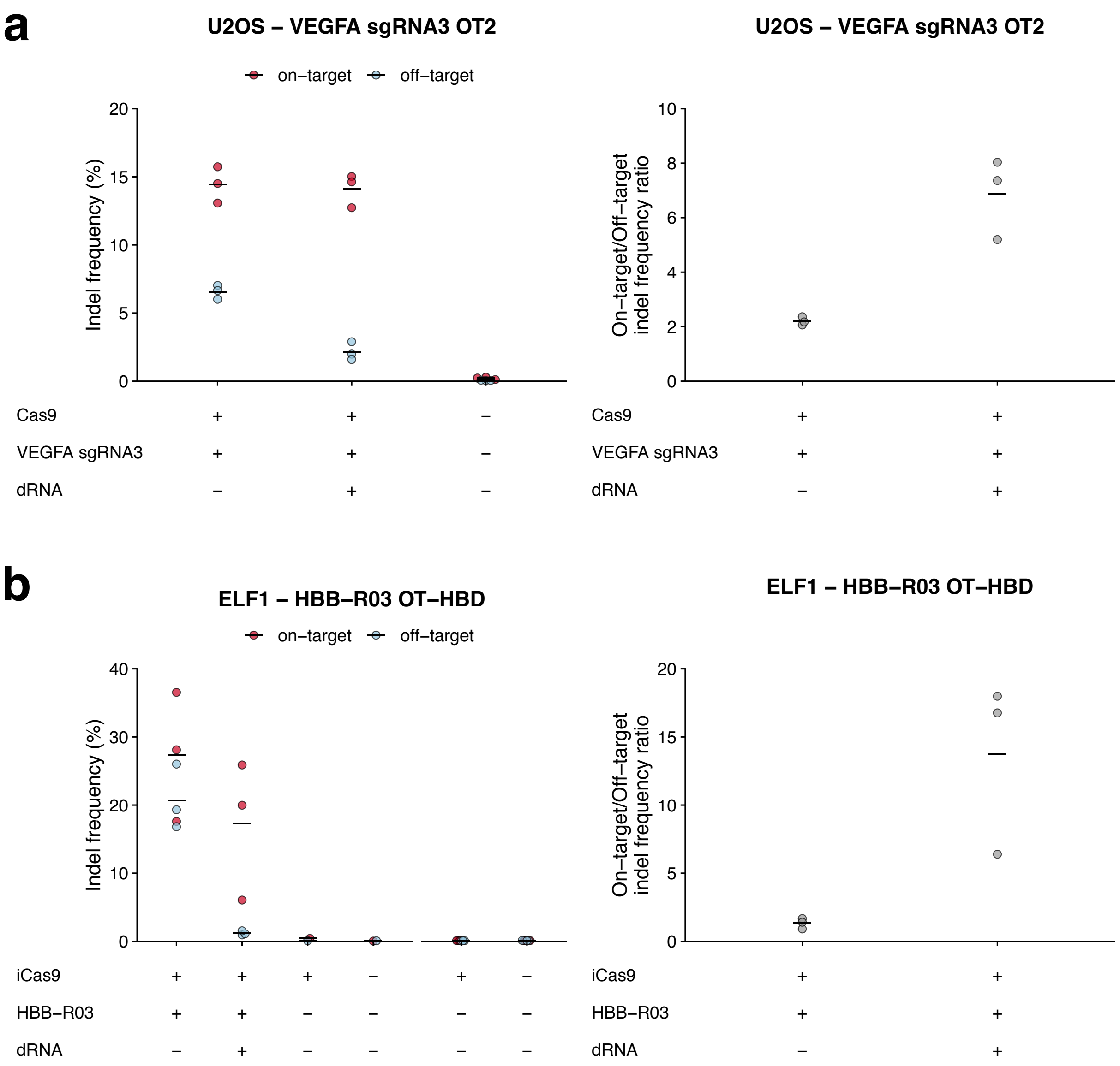

Supplementary Figure 6. dOTS is effective in multiple cell types. On-target and off-target indel frequencies and specificity ratios 24 hours after transfection with Cas9, sgRNA, and off-target specific dRNA. iCas9 denotes stable integration of Cas9 under the control of a doxycycline-inducible promoter. **(a)** *VEGFA* sgRNA3 OT2 in U2OS cells. **(b)** *HBB* R03 OT (HBD) in Elf1 cells. Indel frequencies for untransfected cells are shown as a control. Control samples to the right of the x-axis break were performed separately. Solid lines denote the mean of n = 3 biological replicates. OT = off-target.

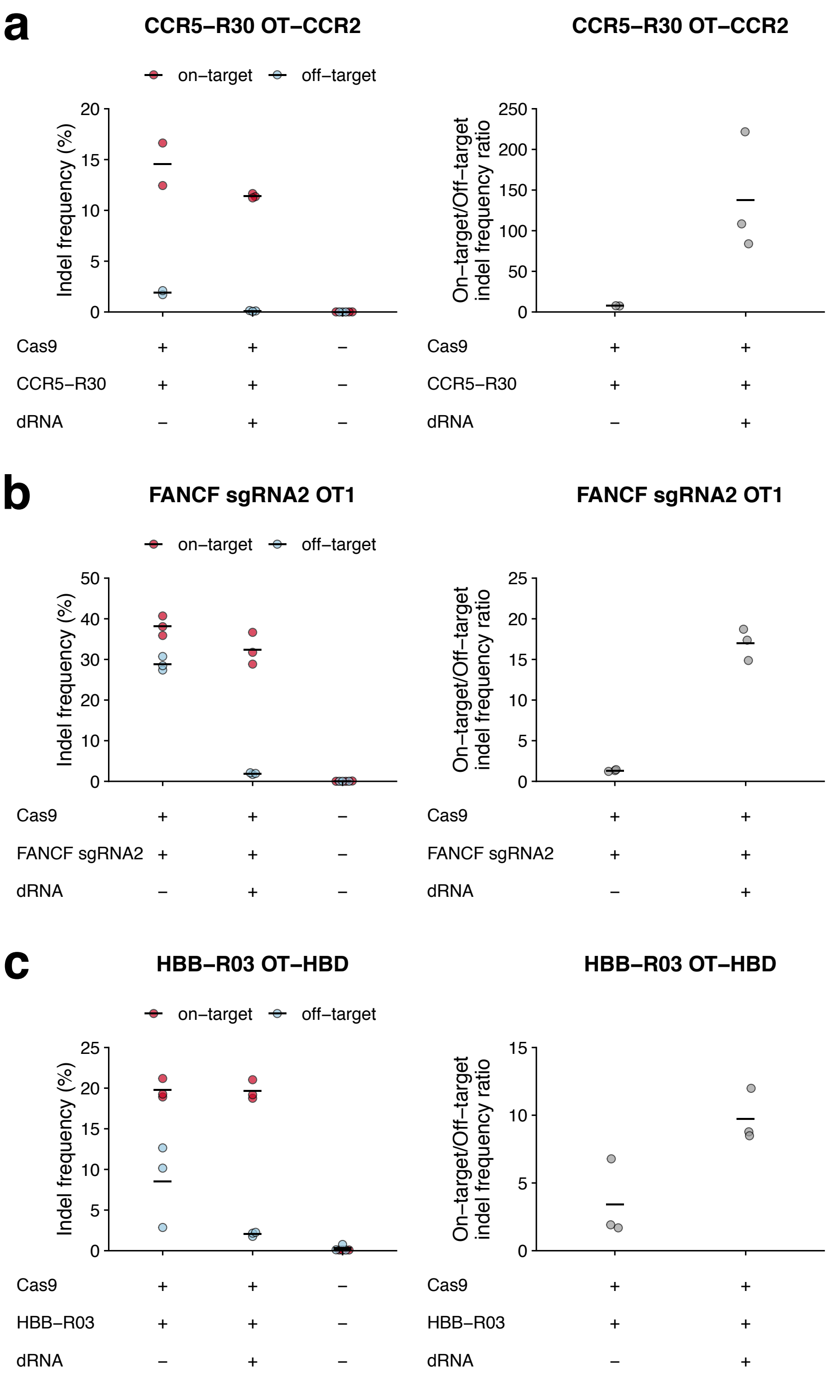

Supplementary Figure 7. dRNA-mediated off-target editing suppression is durable. On-target and off-target indel frequencies and specificity ratios 72 hours after transfection with Cas9, sgRNA, and off-target specific dRNAs in HEK293T cells **(a)** *CCR5*-R30 OT (CCR2). **(b)** *FANCF* sgRNA2 OT1. **(c)** *HBB*-R03 OT (HBD). Indel frequencies for untransfected cells are shown as a control. Numbers denote dRNA identity, see **Supplementary Data Set 1**. Solid lines denote the mean of n = 3 biological replicates, except CCR5-R30 and VEGFA sgRNA3 without dRNA where n = 2. 24 hour comparison shown in **Supplementary Figure 3**.

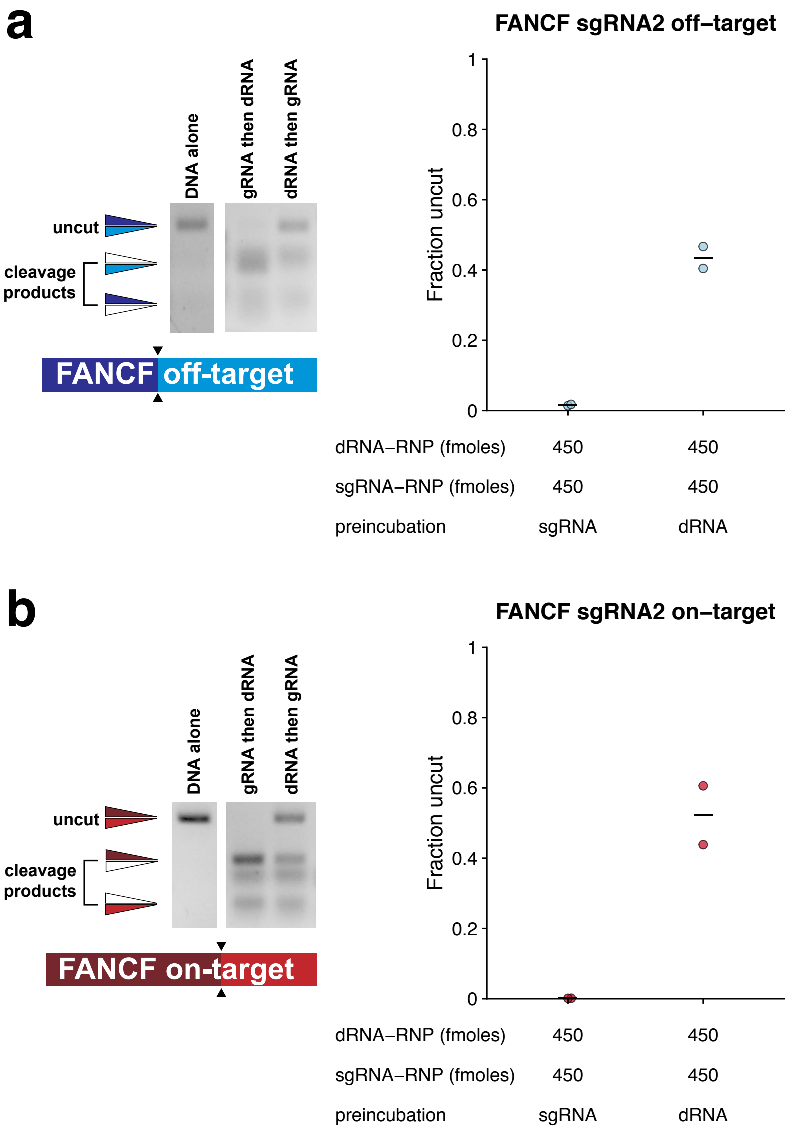

Supplementary Figure 8. dRNAs and sgRNAs compete for target site occupancy. (a, b) Representative gels of *in vitro* Cas9 *FANCF* sgRNA2 RNP cleavage of linear PCR products containing either **(a)** the FANCF sgRNA2 off-target site (OT1) or **(b)** the FANCF sgRNA2 on-target site. PCR products were either preincubated with the sgRNA-RNP complex for 10 minutes prior to addition of the dRNA-RNP complex (sgRNA then dRNA) or were preincubated with the dRNA-RNP complex for 10 minutes prior to addition of the sgRNA-RNP complex (dRNA then sgRNA). ImageJ was used to quantify the intensity of the uncut and all cut bands in each lane. Fraction uncut was determined by dividing uncut intensity by sum of all band intensities in each lane. Solid lines denote the mean of n = 2 biological replicates.

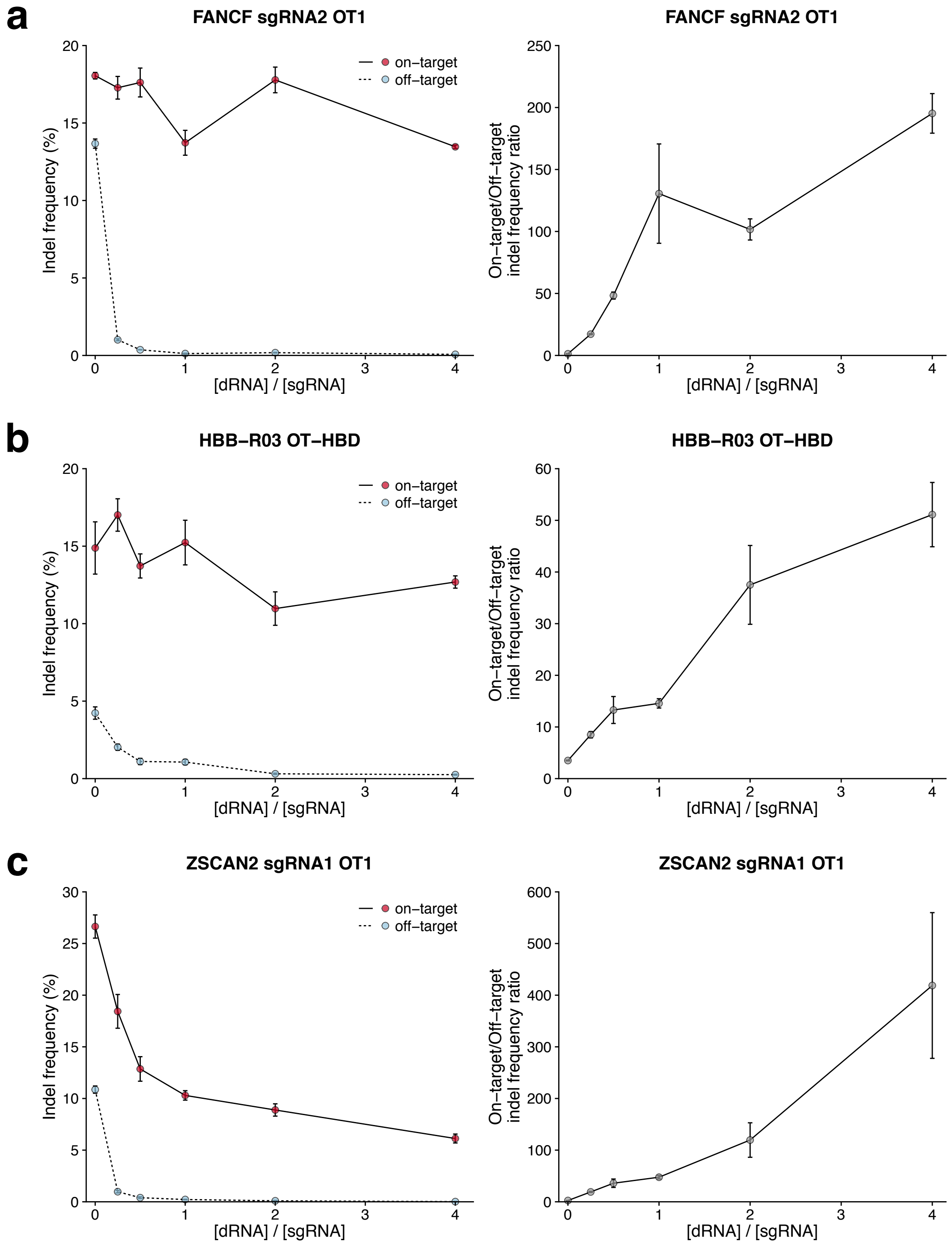

Supplementary Figure 9. Titration of dRNAs further reduces unwanted off-target editing at additional sites. Target and off-target indel frequencies and specificity ratios 24 hours after transfection of various dRNA/sgRNA plasmid ratios for **(a)** FANCF sgRNA 2 and dRNA1; **(b)** *HBB* R03 and dRNA4; **(c)** *ZSCAN2* sgRNA1 and dRNA3. Solid lines denote the mean of n = 3 biological replicates. OT = off-target.

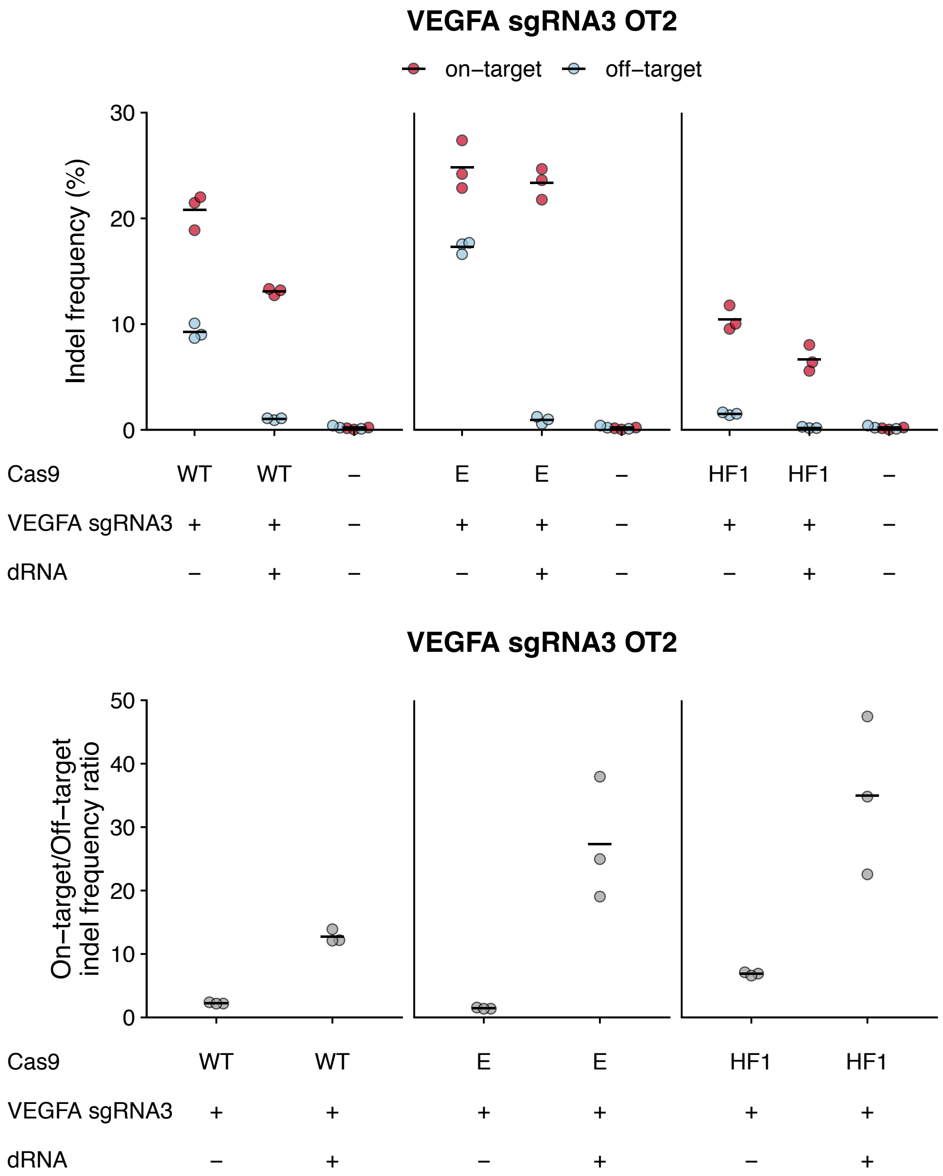

Supplementary Figure 10. dOTS can suppress refractory off-target editing of high-specificity Cas9 variants. On-target and off-target indel frequencies and specificity ratios 24 hours after transfection of plasmids encoding *VEGFA* sgRNA3, dRNA and either wildtype Cas9 (WT), eSpCas9 (E), or SpCas9-HF1 (HF1). Indel frequencies for untransfected cells are shown as a control. Numbers denote dRNA identity, see **Supplementary Data Set 1**. Solid lines denote the mean of n = 3 biological replicates. OT = off-target.

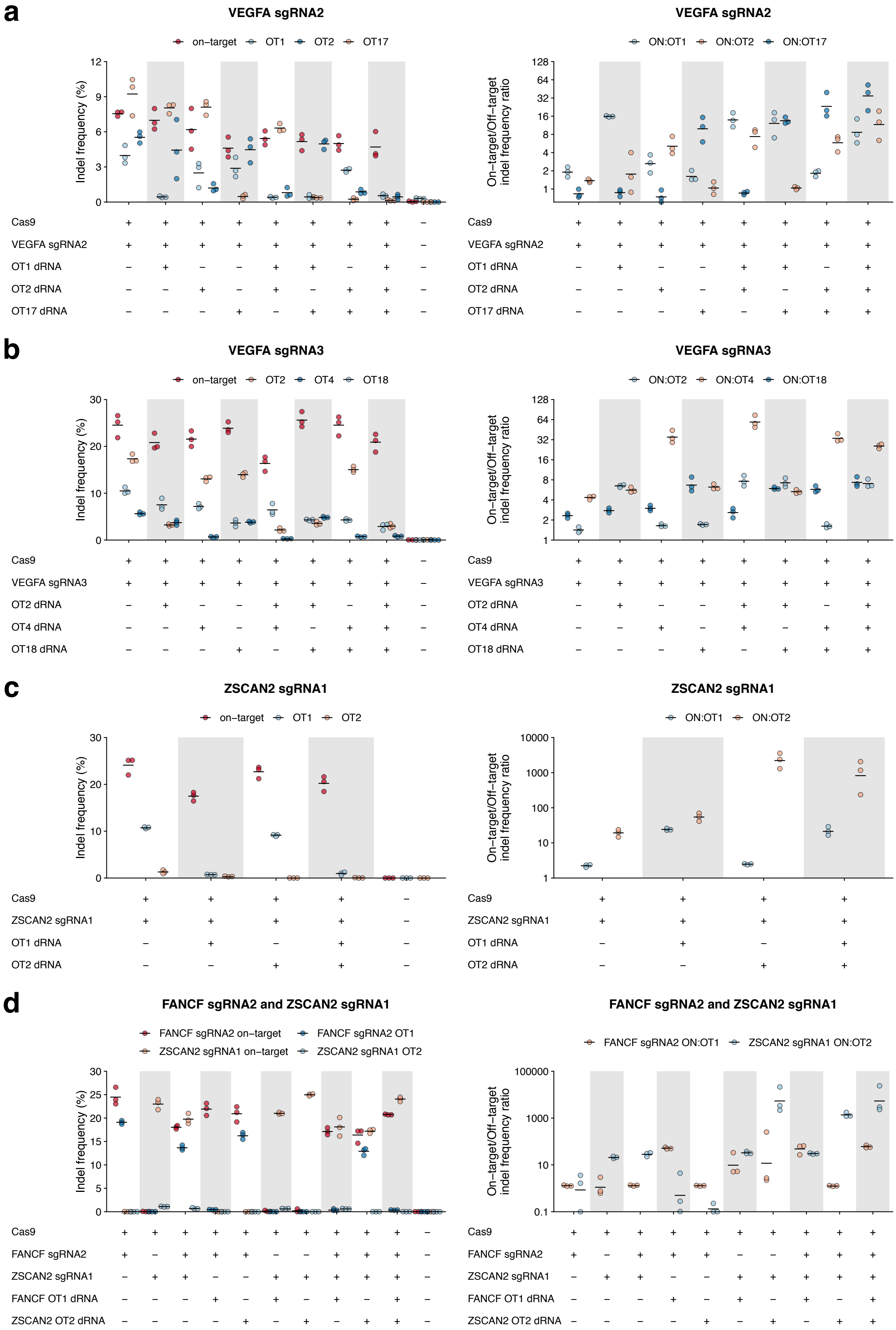

a
VEGFA sgRNA2
on–target
OT1
OT2
OT17
Indel frequency (%)
0
3
6
9
12
Cas9 + + + + + + + + –
VEGFA sgRNA2 + + + + + + + + –
OT1 dRNA – + – – + + – + –
OT2 dRNA – – + – + – + + –
OT17 dRNA – – – + – + + + –
VEGFA sgRNA2
ON:OT1
ON:OT2
ON:OT17
On–target/Off–target indel frequency ratio
1
2
4
8
16
32
64
128
Cas9 + + + + + + + +
VEGFA sgRNA2 + + + + + + + +
OT1 dRNA – + – – + + – +
OT2 dRNA – – + – + – + +
OT17 dRNA – – – + – + + +
b
VEGFA sgRNA3
on–target
OT2
OT4
OT18
Indel frequency (%)
0
10
20
30
Cas9 + + + + + + + + –
VEGFA sgRNA3 + + + + + + + + –
OT2 dRNA – + – – + + – + –
OT4 dRNA – – + – + – + + –
OT18 dRNA – – – + – + + + –
VEGFA sgRNA3
ON:OT2
ON:OT4
ON:OT18
On–target/Off–target indel frequency ratio
1
2
4
8
16
32
64
128
Cas9 + + + + + + + +
VEGFA sgRNA3 + + + + + + + +
OT2 dRNA – + – – + + – +
OT4 dRNA – – + – + – + +
OT18 dRNA – – – + – + + +
c
ZSCAN2 sgRNA1
on–target
OT1
OT2
Indel frequency (%)
0
10
20
30
Cas9 + + + + –
ZSCAN2 sgRNA1 + + + + –
OT1 dRNA – + – + –
OT2 dRNA – – + + –
ZSCAN2 sgRNA1
ON:OT1
ON:OT2
On–target/Off–target indel frequency ratio
1
10
100
1000
10000
Cas9 + + + +
ZSCAN2 sgRNA1 + + + +
OT1 dRNA – + – +
OT2 dRNA – – + +
d
FANCF sgRNA2 and ZSCAN2 sgRNA1
FANCF sgRNA2 on–target
FANCF sgRNA2 OT1
ZSCAN2 sgRNA1 on–target
ZSCAN2 sgRNA1 OT2
Indel frequency (%)
0
5
10
15
20
25
30
Cas9 + + + + + + + + + + –
FANCF sgRNA2 + – + + + – – + + + –
ZSCAN2 sgRNA1 – + + – – + + + + + –
FANCF OT1 dRNA – – – + – + – + – + –
ZSCAN2 OT2 dRNA – – – – + – + – + + –
FANCF sgRNA2 and ZSCAN2 sgRNA1
FANCF sgRNA2 ON:OT1
ZSCAN2 sgRNA1 ON:OT2
On–target/Off–target indel frequency ratio
0.1
10
1000
100000
Cas9 + + + + + + + + + +
FANCF sgRNA2 + – + + + – – + + +
ZSCAN2 sgRNA1 – + + – – + + + + +
FANCF OT1 dRNA – – – + – + – + – +
ZSCAN2 OT2 dRNA – – – – + – + – + +

Supplementary Figure 11. dRNA can be combined to suppress unwanted off-target editing at a variety of sites. Target and off-target indel frequencies and specificity ratios 24 hours after transfection with plasmids encoding Cas9 and various combinations of sgRNAs and dRNAs at **(a)** *VEGFA* sgRNA2 OT1, OT2, and OT17; **(b)** *VEGFA* sgRNA3 OT2, OT4, and OT18; **(c)** *ZSCAN2* sgRNA1 OT1 and OT2; **(d)** *FANCF* sgRNA2 OT1 and *ZSCAN2* sgRNA1 OT2. Indel frequencies for untransfected cells are shown as a control. Solid lines denote the mean of n = 3 biological replicates. OT = off-target.

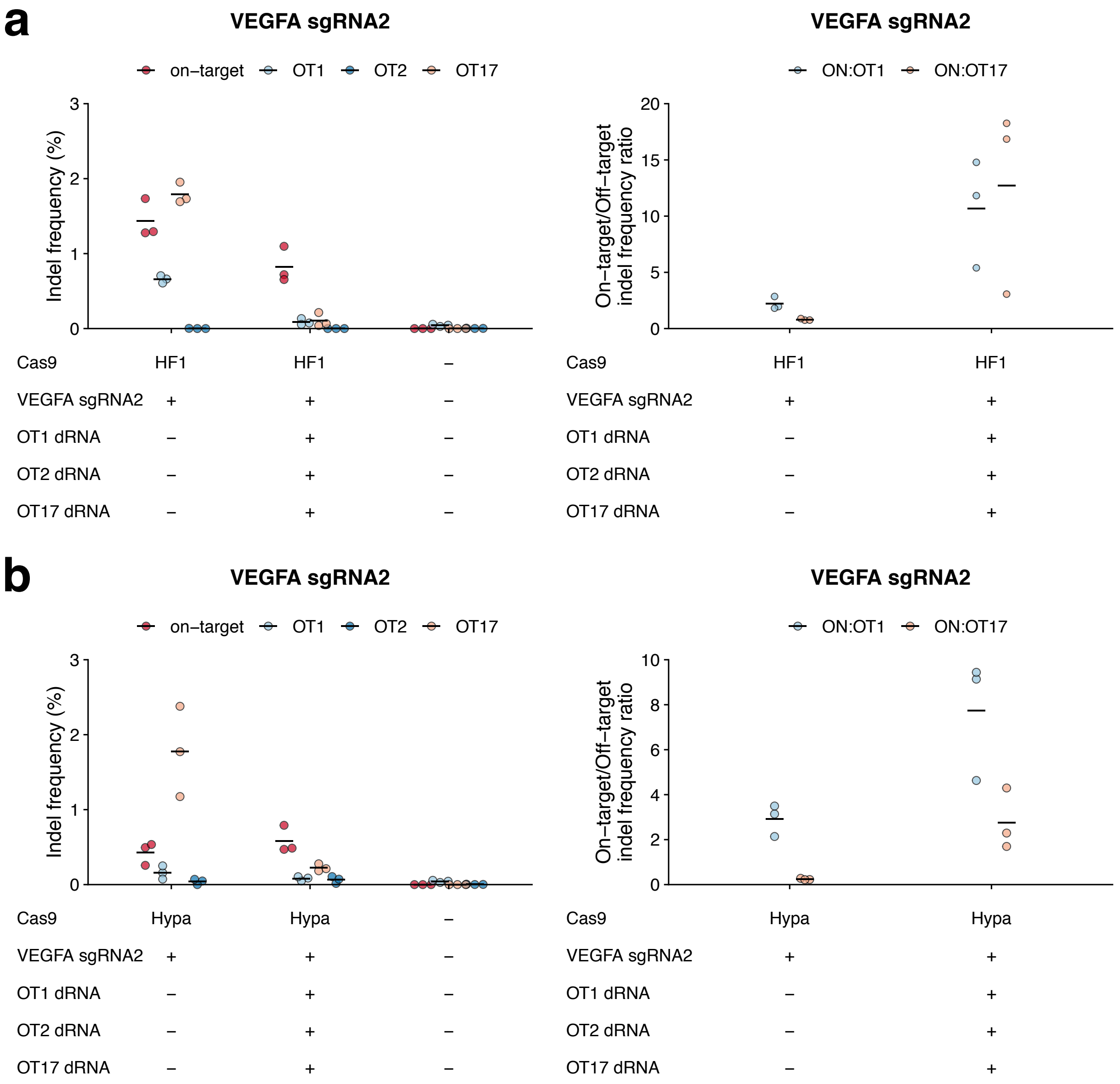

Supplementary Figure 12. Multiple dRNAs can be combined to reduce unwanted editing at multiple refractory off-target sites of high-specificity Cas9 variants. Target and off-target indel frequencies and specificity ratios 24 hours after transfection with plasmids encoding *VEGFA* sgRNA2, a combination of three dRNAs and either **(a)** SpCas9-HF1 (HF1) or **(b)** HypaCas9 (Hypa). Despite being reported previously[21], indels were not observed at OT2, so specificity ratios were not plotted. Indel frequencies for untransfected cells are shown as a control. Solid lines denote the mean of n = 3 biological replicates. OT = off-target.

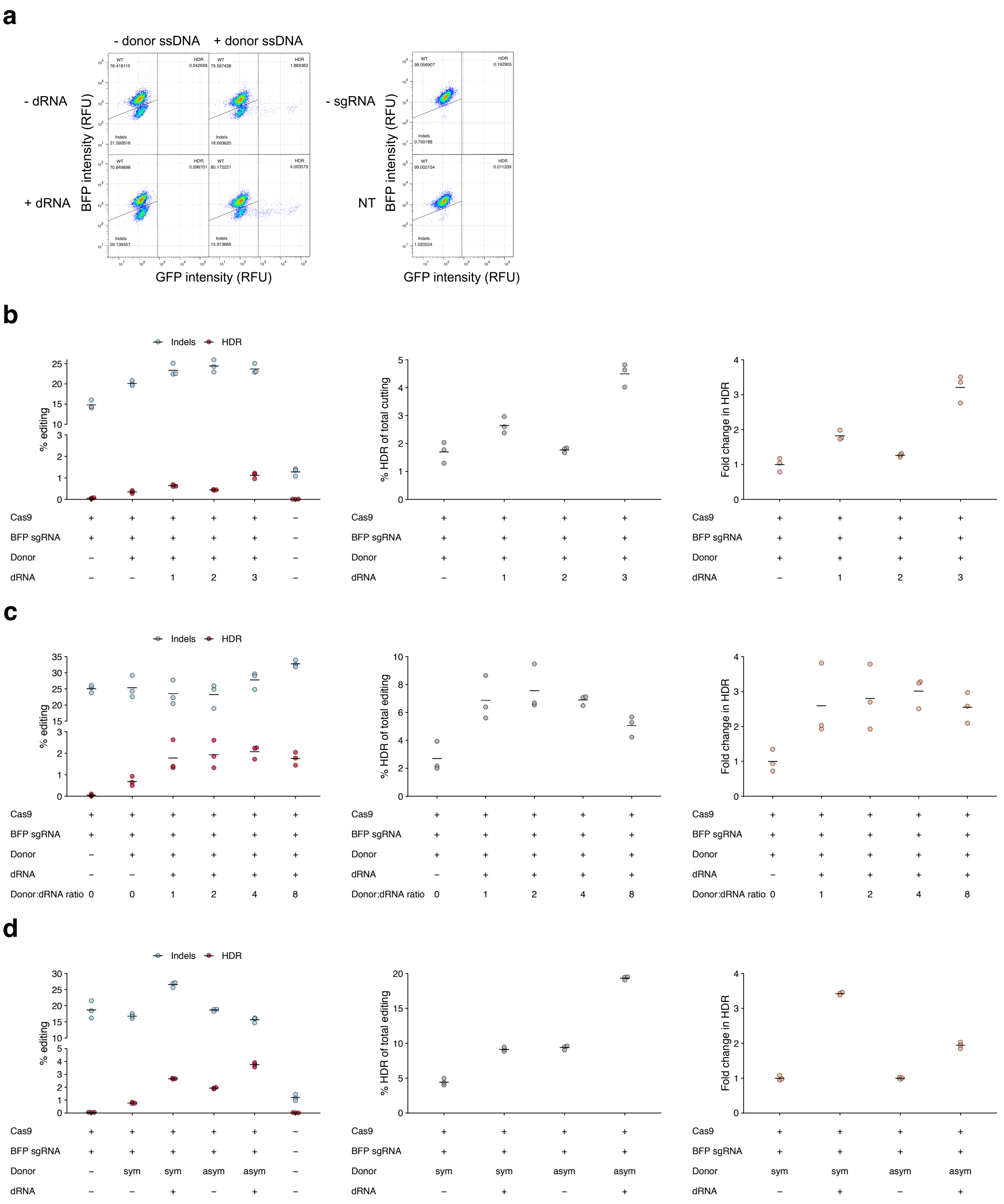

Supplementary Figure 13. Screening guides, donors, and dRNAs for scarless HDR in a fluorescent reporter system. (a) Representative flow cytometry plots illustrating alteration of a synthetic BFP locus to GFP after Cas9•sgRNA editing. **(b)** Screening of three dRNAs for indels or HDR events, percent HDR of total Cas9 editing observed, and fold change in HDR observed. **(c)** Screening of various ratios of dRNA3 to sgRNA for indels or HDR events, percent HDR of total Cas9 editing observed, and fold change in HDR observed. **(d)** Comparison of symmetric donor and asymmetric donor for indels or HDR events, percent HDR of total Cas9 editing observed, and fold change in HDR observed. HDR donors do not contain blocking mutations. Indel frequencies for untransfected cells are shown as a control. Numbers denote sgRNA and dRNA identities, see **Supplementary Data Set 1**. Solid lines denote the mean of n = 3 biological replicates.

FANCF sgRNA2 - Replicate 1

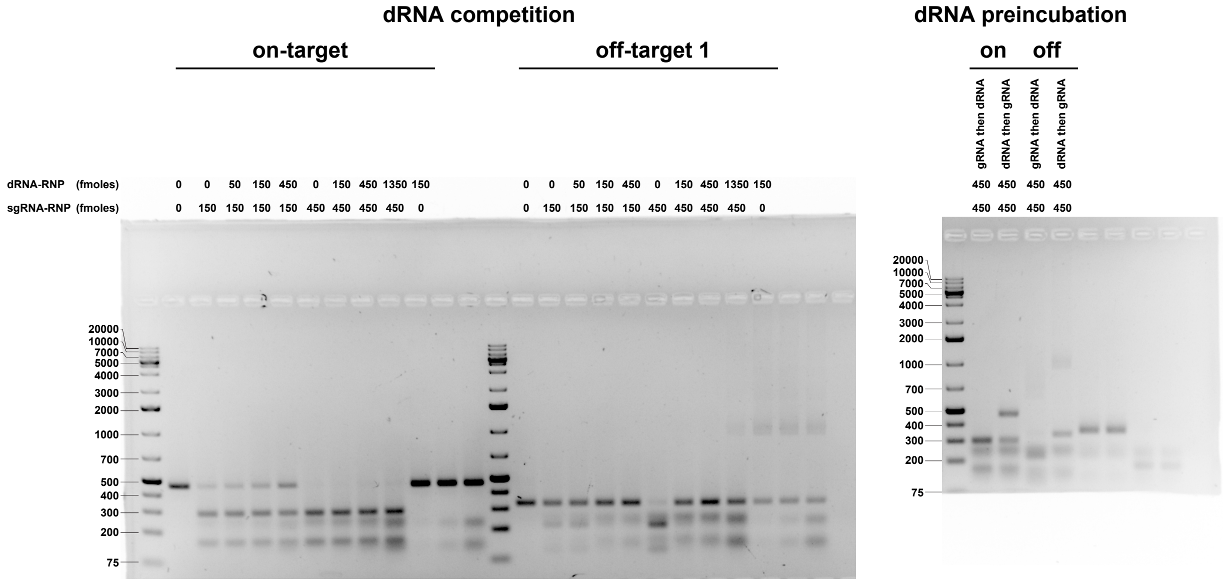

FANCF sgRNA2 - Replicate 2

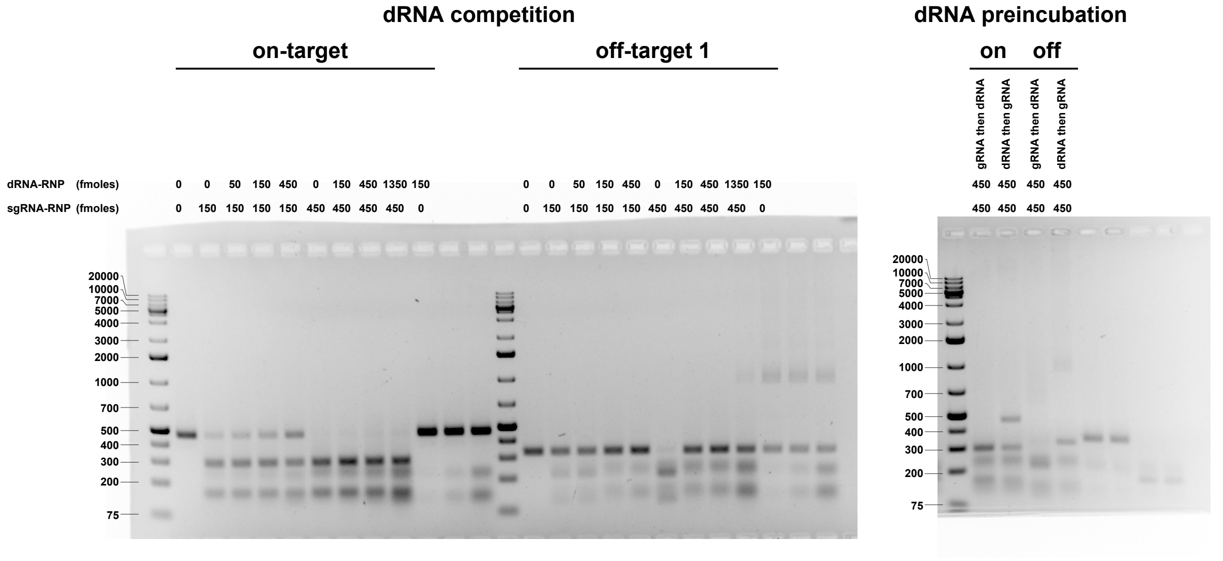

Supplementary Figure 14. Uncropped gel images for Figure 2 and Supplementary Figure 8. Full gel images of *in vitro* Cas9 *FANCF* sgRNA2 RNP cleavage of linear PCR products containing either the *FANCF* sgRNA2 on- or off-target site (OT1).

Supplementary Tables

Site	Best dRNA	n	Normalized specificity ratio (mean)	On-target ratio (mean)	Normalized specificity ratio (s.e.m)	On-target ratio (s.e.m)
***ZSCAN2* sgRNA1 OT2**	1s	3	37.93	0.73	7.07	0.04
***FANCF* sgRNA2 OT1**	1	3	29.96	1.04	3.42	0.04
***VEGFA* sgRNA2 OT17**	8	3	13.11	1.02	1.57	0.08
***CCR5*-R30 OT-CCR2**	3	3	11.34	0.57	6.81	0.28
***ZSCAN2* sgRNA1 OT1**	3	3	8.95	0.82	2.07	0.04
***VEGFA* sgRNA2 OT2**	8	3	7.50	1.00	2.60	0.21
HBB*-R01 OT-*HBD	2	3	7.48	0.89	1.74	0.18
***VEGFA* sgRNA3 OT4**	1	3	6.75	1.48	1.68	0.07
***VEGFA* sgRNA1 OT1**	2	3	6.72	0.93	0.97	0.10
HBB*-R03 OT-*HBD	4	3	6.55	0.89	2.01	0.12
***VEGFA* sgRNA1 OT4**	8	3	4.99	0.51	1.37	0.14
***VEGFA* sgRNA1 OT6**	8	3	4.57	0.66	1.49	0.19
***VEGFA* sgRNA2 OT1**	1	3	4.32	1.05	0.92	0.10
***VEGFA* sgRNA3 OT2**	2	3	4.26	1.04	0.43	0.04
***VEGFA* sgRNA3 OT18**	5	3	2.13	1.33	1.13	0.50
***VEGFA* sgRNA1 OT11**	7	3	40.60	0.31	16.94	0.08
***HBB*-G10 OT1**	7	3	3.74	0.47	1.49	0.08
***VEGFA* sgRNA2 OT19**	5	3	1.55	0.72	0.55	0.13
HBB*-R04 OT-*HBD	4	3	1.16	0.77	0.29	0.09

Supplementary Table 1. dRNAs designed for a variety of sites increase specificity ratio with minimal effects on on-target editing. Normalized specificity ratios, computed as the specificity ratio in the presence of the best dRNA at a site divided by the specificity ratio in the absence of

the dRNA, and on-target ratios, computed as the ratio of on-target editing in the presence of the best dRNA at a site divided by the on-target editing in the absence of the dRNA, for the best dRNA for 19 sgRNA/off-target pairs. n = 3 biological replicates, error measured as the standard error of the mean (s.e.m.).

s

**Site**	**Δ (On)**	**p (On)**	**p_{adj} (On)**	**Δ (OT)**	**p (OT)**	**p_{adj} (OT)**
***FANCF* sgRNA2 OT1**	-0.004	0.835	1	-0.002	0.910	1
HBB* R03 OT-*HBD	-0.014	0.845	1	-0.009	0.761	1
***VEGFA* sgRNA1 OT1**	6.07E-04	0.403	1	6.61E-04	0.094	1
***VEGFA* sgRNA1 OT6**	-1.95E-04	0.524	1	0.025	0.209	1
***VEGFA* sgRNA2 OT1**	-0.045	0.912	1	0.083	0.124	1
***VEGFA* sgRNA2 OT2**	-0.018	0.750	1	-9.37E-04	0.683	1
***VEGFA* sgRNA2 OT17**	0.015	0.306	1	0.008	0.218	1
***VEGFA* sgRNA3 OT4**	-0.007	0.907	1	0	1	1
***VEGFA* sgRNA3 OT18**	-3.27E-04	0.513	1	0.002	0.319	1
***ZSCAN2* sgRNA1 OT1**	-3.87E-04	0.789	1	0	1	1
***ZSCAN2* sgRNA1 OT2**	3.15E-05	0.479	1	0.001	0.092	1
***VEGFA* sgRNA3 OT2**	0.040	0.406	1	0.080	0.050	1

Supplementary Table 2. dRNAs alone do not promote editing at sgRNA target sites. Difference between indel frequencies at on- and off-target (OT) sites for the best dRNA compared to a negative control at 12 different on/off-target pairs (Δ). p: p-value, based on two-sided Student's t-test. $p_{adj:}$ Bonferroni-adjusted p-value. n = 3 biological replicates at on- and off-target sites, except for *VEGFA* sgRNA3 OT2 (n = 9) and *VEGFA* sgRNA3 OT18 (n = 3 at on-target, n = 2 at off-target due to failed sequencing reactions).

Site	Δ (On$_{pred}$)	p (On$_{pred}$)	p_{adj} (On$_{pred}$)	Δ (OT$_{pred}$)	p (OT$_{pred}$)	p_{adj} (OT$_{pred}$)
FANCF **sgRNA2 OT1**	-0.004	0.835	1	-0.002	0.910	1
HBB **R03 OT-*HBD***	-0.056	0.699	1	-0.006	0.828	1
VEGFA **sgRNA1 OT1**	-0.004	0.730	1	0.001	0.312	1
VEGFA **sgRNA1 OT6**	7.78E-04	0.278	1	0.027	0.204	1
VEGFA **sgRNA2 OT1**	0.316	0.220	1	0.083	0.124	1
VEGFA **sgRNA2 OT2**	0.028	0.253	1	0.001	0.302	1
VEGFA **sgRNA2 OT17**	0.092	0.115	1	0.074	0.260	1
VEGFA **sgRNA3 OT4**	-0.015	0.908	1	0	1	1
VEGFA **sgRNA3 OT18**	6.55E-05	0.471	1	-0.013	0.622	1
ZSCAN2 **sgRNA1 OT1**	0.002	0.089	1	-7.37E-04	0.539	1
ZSCAN2 **sgRNA1 OT2**	3.15E-05	0.479	1	0.001	0.092	1
VEGFA **sgRNA3 OT2**	0.061	0.094	1	0.073	0.071	1

Supplementary Table 3. dRNAs alone do not promote editing at predicted dRNA target sites. Difference between indel frequencies at on- and off-target (OT) sites for the best dRNA compared to a negative control at 12 different on/off-target pairs (Δ). Predicted indel locations (pred) are the location of expected indels if the dRNA were a full length sgRNA. p: p-value, based on two-sided Student's t-test. $p_{adj:}$ Bonferroni-adjusted p-value. n = 3 biological replicates at on- and off-target sites, except for *VEGFA* sgRNA3 OT2 (n = 9) *VEGFA* sgRNA2 OT17 (n = 3 at on-target, n = 2 at off-target due to failed sequencing reactions), and *VEGFA* sgRNA3 OT18 (n = 3 at on-target, n = 2 at off-target due to failed sequencing reactions).
