# Supplementary material for: Suppression of unwanted CRISPR-Cas9 editing by co-administration of catalytically inactivating truncated guide RNAs
Source: Nat Commun. 2020 Jun 1;11:2697. doi: 10.1038/s41467-020-16542-9 (PMC7264211; doi:10.1038/s41467-020-16542-9)
Supplement: Supplementary file 3 — Reporting Summary [file 41467_2020_16542_MOESM3_ESM.pdf]

nature research

Corresponding author(s): Douglas Fowler

Reporting Summary

Nature Research wishes to improve the reproducibility of the work that we publish. This form provides structure for consistency and transparency in reporting. For further information on Nature Research policies, see Authors & Referees and the Editorial Policy Checklist.

Statistical parameters

When statistical analyses are reported, confirm that the following items are present in the relevant location (e.g. figure legend, table legend, main text, or Methods section).

n/a	Confirmed	
☐	☒	The exact sample size (*n*) for each experimental group/condition, given as a discrete number and unit of measurement
☐	☒	An indication of whether measurements were taken from distinct samples or whether the same sample was measured repeatedly
☐	☒	The statistical test(s) used AND whether they are one- or two-sided<br>*Only common tests should be described solely by name; describe more complex techniques in the Methods section.*
☒	☐	A description of all covariates tested
☐	☒	A description of any assumptions or corrections, such as tests of normality and adjustment for multiple comparisons
☐	☒	A full description of the statistics including central tendency (e.g. means) or other basic estimates (e.g. regression coefficient) AND variation (e.g. standard deviation) or associated estimates of uncertainty (e.g. confidence intervals)
☐	☒	For null hypothesis testing, the test statistic (e.g. *F*, *t*, *r*) with confidence intervals, effect sizes, degrees of freedom and *P* value noted<br>*Give P values as exact values whenever suitable.*
☒	☐	For Bayesian analysis, information on the choice of priors and Markov chain Monte Carlo settings
☒	☐	For hierarchical and complex designs, identification of the appropriate level for tests and full reporting of outcomes
☒	☐	Estimates of effect sizes (e.g. Cohen's *d*, Pearson's *r*), indicating how they were calculated
☐	☒	Clearly defined error bars<br>*State explicitly what error bars represent (e.g. SD, SE, CI)*

Our web collection on statistics for biologists may be useful.

Software and code

Policy information about availability of computer code

Data collection	High throughput sequencing reads were demultiplexed using bcl2fastq/2.18 (Illumina). Flow cytometry samples were collected using BD FACSDiva v. 8.0.1
Data analysis	Demultiplexed reads were then analyzed for indels using a custom Python (v. 2.7) script using the difflib package (v. 2.3) and is available upon request. Flow cytometry data was analyzed using FlowJo (v. 10.4.2). Figures and other data visualization was performed in R (v. 3.4.4). Data were analysed with the publicly available GUIDE-seq software allowing for up to 8 mismatches with a modification of a 35 bp window for detected off-target alignments to reference sequence.

For manuscripts utilizing custom algorithms or software that are central to the research but not yet described in published literature, software must be made available to editors/reviewers upon request. We strongly encourage code deposition in a community repository (e.g. GitHub). See the Nature Research guidelines for submitting code & software for further information.

Data

Policy information about availability of data

All manuscripts must include a data availability statement. This statement should provide the following information, where applicable:

- Accession codes, unique identifiers, or web links for publicly available datasets
- A list of figures that have associated raw data
- A description of any restrictions on data availability

Raw sequencing data will be made available upon publication through the NCBI GEO repository. All figures (1-6 and S1-S10) have associated raw data.

Field-specific reporting

Please select the best fit for your research. If you are not sure, read the appropriate sections before making your selection.

☒ Life sciences ☐ Behavioural & social sciences ☐ Ecological, evolutionary & environmental sciences

For a reference copy of the document with all sections, see nature.com/authors/policies/ReportingSummary-flat.pdf

Life sciences study design

All studies must disclose on these points even when the disclosure is negative.

Sample size	Sample size calculations were not performed. Sample sizes were chosen to detect the large effects we discuss in the paper.
Data exclusions	Some data were excluded from the analysis because of repeated failed high throughput sequencing reactions (< 1000 filtered reads) and are noted in the text and figure legends where appropriate.
Replication	All experiments were performed with at least 3 biological replicates per condition.
Randomization	Randomization is not applicable. Each experiment contained millions of isogenic cells and there was no variation before treatment.
Blinding	All data analysis was performed on blinded samples.

Reporting for specific materials, systems and methods

Materials & experimental systems

n/a	Involved in the study	
☒	☐	Unique biological materials
☒	☐	Antibodies
☐	☒	Eukaryotic cell lines
☒	☐	Palaeontology
☒	☐	Animals and other organisms
☒	☐	Human research participants

Methods

n/a	Involved in the study	
☒	☐	ChIP-seq
☐	☒	Flow cytometry
☒	☐	MRI-based neuroimaging

Eukaryotic cell lines

Policy information about cell lines

Cell line source(s)	HEK293T/17: ATCC. U2OS: ATCC. hESC Elf1: developed by co-author (NIH_hESC Registry #0156)
Authentication	Cells were not authenticated.
Mycoplasma contamination	All cell lines were tested negative for mycoplasma.
Commonly misidentified lines (See ICLAC register)	No commonly misidentified cell lines were used.

Flow Cytometry

Plots

Confirm that:

- [x] The axis labels state the marker and fluorochrome used (e.g. CD4-FITC).
- [x] The axis scales are clearly visible. Include numbers along axes only for bottom left plot of group (a 'group' is an analysis of identical markers).
- [x] All plots are contour plots with outliers or pseudocolor plots.
- [x] A numerical value for number of cells or percentage (with statistics) is provided.

Methodology

Sample preparation	HEK293T cells (ATCC) were washed with 2 mL DPBS, trypsinized with 0.5 mL 0.25% trypsin-EDTA (Life Technologies) for 2-4 minutes, and quenched with DMEM supplemented with 10% FBS. Cells were then spun down at 290 x g for 4 min, aspirated, and resuspended in DPBS supplemented with 1% FBS. Cells were passed through a 35 µm filter and analyzed by flow cytometry on an LSR-II flow cytometer.
Instrument	BD LSR II special order system, part number 640752, serial number H33910100001
Software	All data were collected using BD FACSDiva v. 8.0.1 and analyzed using FlowJo v. 10.4.2. Summary figure plots were generated using R v. 3.4.4.
Cell population abundance	Each sample was screened for 10,000 live cell events and analyzed using FlowJo v. 10.4.2. Cells were not used after sorting.
Gating strategy	Live cells were first gated on FSC-A x SSC-A, then subjected to a FSC-A x SSC-W to select for single cells. Cells were then gated on BFP x GFP (BFP: violet laser (405 nm, 100 mW) with 450/50 band pass filter; GFP: blue laser (488 nm, 100 mW) with 530/30, 505LP band pass filter.) Populations were classified in accordance to the methods in Richardson, et al. Nature Biotechnology, 2016. A negative (BFP+, GFP-; no editing) population was determined using a cell line that was not transfected. An NHEJ (BFP-, GFP-) population was determined using cells transfected with Cas9 and a sgRNA targeting the BFP locus. An HDR population (GFP +) population was determined as any cells exhibiting increased GFP fluorescence compared to these two controls.

- [x] Tick this box to confirm that a figure exemplifying the gating strategy is provided in the Supplementary Information.
