# Supplementary material for: Suppression of unwanted CRISPR-Cas9 editing by co-administration of catalytically inactivating truncated guide RNAs
Source: Nat Commun. 2020 Jun 1;11:2697. doi: 10.1038/s41467-020-16542-9 (PMC7264211; doi:10.1038/s41467-020-16542-9)
Supplement: Supplementary file 4 — Description of Additional Supplementary Files [file 41467_2020_16542_MOESM4_ESM.pdf]

Title: Supplementary data file 1:

Description: Primers, amplicons, sgRNA and dRNA templates, hg38 genomic locations, and HDR donors used in this study.
